# Diagnostic Utility of 18F‐Florzolotau PET Imaging in Patients with Cognitive Symptoms

**DOI:** 10.1002/alz70856_102537

**Published:** 2025-12-25

**Authors:** Mei Cui, Jiawei Xin, Yu Guo, Ziyi Wang, Jiaying Lu, Yu‐Yuan Huang, Chuantao Zuo, Qianhua Zhao, Jin‐Tai Yu

**Affiliations:** ^1^ MOE Frontiers Center for Brain Science, Fudan University, Shanghai, China; ^2^ Huashan Hospital, Fudan University, Shanghai, Shanghai, China; ^3^ Department of Neurology, Fujian Medical University Affiliate Union Hospital, Fuzhou, China; ^4^ Department of Nuclear Medicine and PET Center, Huashan Hospital, Fudan University, Shanghai, Shanghai, China; ^5^ Department of Neurology and National Center for Neurological Disorders, Huashan Hospital, State Key Laboratory of Medical Neurobiology and MOE Frontiers Center, Fudan University, Shanghai, Shanghai, China; ^6^ National Center for Neurological Disorders, Shanghai, China

## Abstract

**Background:**

The clinical utility of 18F‐Florzolotau PET imaging in evaluating unselected patients presenting with cognitive complaints in a real‐world memory clinic setting remains unclear. This cohort study was designed to comprehensively evaluate the diagnostic accuracy, reclassification impact, and influence on diagnostic confidence of 18F‐Florzolotau PET across various cognitive status groups and neurodegenerative conditions.

**Method:**

A total of 838 cognitively impaired patients underwent both amyloid PET and 18F‐Florzolotau PET scans.

**Result:**

Compared to final diagnoses, 18F‐Florzolotau PET achieved a high degree of diagnostic accuracy for both primary (progressive supranuclear palsy, 80%; corticobasal syndrome, 75%) and secondary (Alzheimer's disease [AD], 95.7%) tauopathies. For AD diagnosis, 18F‐Florzolotau PET exhibited a sensitivity of 98.2% and specificity of 96.7%, surpassing routine diagnostic procedures. Within the AD spectrum, its performance was comparable to Aβ PET across various cognitive stages, including subjective cognitive decline (SCD), mild cognitive impairment (MCI), and dementia. Notably, diagnostic reclassifications prompted by 18F‐Florzolotau PET findings were significantly greater in the SCD (36.8%) and MCI (30.6%) groups compared to dementia (16.4%, both *p* < 0.001). In non‐AD tauopathies, the tracer modified diagnoses in 45.5% of cases and significantly impacted the final patient classification. Furthermore, diagnostic confidence improved markedly from 68.6% to 84.2% after 18F‐Florzolotau PET, exceeding that obtained with amyloid PET alone (*p* < 0.001).

**Conclusion:**

In conclusion, 18F‐Florzolotau PET exhibits substantial potential for the early and accurate detection of primary and secondary tauopathies. Its integration into real‐world practice could significantly improve the diagnostic workflow of cognitive disorders, aligning with the critical roles of memory clinics in providing high‐quality assessments.

